# Autonomic and Brain Morphological Predictors of Stress Resilience

**DOI:** 10.3389/fnins.2018.00228

**Published:** 2018-04-06

**Authors:** Luca Carnevali, Julian Koenig, Andrea Sgoifo, Cristina Ottaviani

**Affiliations:** ^1^Neuroimaging Laboratory, Santa Lucia Foundation, Rome, Italy; ^2^Stress Physiology Lab, Department of Chemistry, Life Sciences and Environmental Sustainability, University of Parma, Parma, Italy; ^3^Section for Translational Psychobiology in Child and Adolescent Psychiatry, Department of Child and Adolescent Psychiatry, Centre for Psychosocial Medicine, University of Heidelberg, Heidelberg, Germany; ^4^University Hospital of Child and Adolescent Psychiatry and Psychotherapy, University of Bern, Bern, Switzerland; ^5^Department of Psychology, Sapienza University of Rome, Rome, Italy

**Keywords:** anterior cingulate cortex, cortical thickness, depression, heart rate variability, PTSD, resilience, stress

## Abstract

Stressful life events are an important cause of psychopathology. Humans exposed to aversive or stressful experiences show considerable inter-individual heterogeneity in their responses. However, the majority does not develop stress-related psychiatric disorders. The dynamic processes encompassing positive and functional adaptation in the face of significant adversity have been broadly defined as *resilience*. Traditionally, the assessment of resilience has been confined to self-report measures, both within the general community and putative high-risk populations. Although this approach has value, it is highly susceptible to subjective bias and may not capture the dynamic nature of resilience, as underlying construct. Recognizing the obvious benefits of more objective measures of resilience, research in the field has just started investigating the predictive value of several potential biological markers. This review provides an overview of theoretical views and empirical evidence suggesting that individual differences in heart rate variability (HRV), a surrogate index of resting cardiac vagal outflow, may underlie different levels of resilience toward the development of stress-related psychiatric disorders. Following this line of thought, recent studies describing associations between regional brain morphometric characteristics and resting state vagally-mediated HRV are summarized. Existing studies suggest that the structural morphology of the anterior cingulated cortex (ACC), particularly its cortical thickness, is implicated in the expression of individual differences in HRV. These findings are discussed in light of emerging structural neuroimaging research, linking morphological characteristics of the ACC to psychological traits ascribed to a high-resilient profile and abnormal structural integrity of the ACC to the psychophysiological expression of stress-related mental health consequences. We conclude that a multidisciplinary approach integrating brain structural imaging with HRV monitoring could offer novel perspectives about brain-body pathways in resilience and adaptation to psychological stress.

## Introduction

Psychological stress is common in our fast-paced society and strongly influences our mental and physical well-being. Humans exposed to common aversive or stressful experiences exhibit a wide range of responses, yet the majority maintains normative psychological and physiological functioning. The ability of an individual to withstand and adapt to stress and adversity is broadly defined as *resilience* (Fletcher and Sarkar, 2013; Horn et al., 2016). The question as to why resilient individuals minimize pathophysiological outcomes when faced with stressful life events, while others succumb to stress and develop psychopathologies such as anxiety, depression, and post-traumatic stress disorder (PTSD), as well as somatic ill-health, has been addressed by scientists for decades, resulting in a fairly comprehensive description of the psychosocial factors linked to a stress resilient profile. The use of adaptive coping strategies, the individual emotion regulatory capacity, factors like optimism, and an ability to face fears, as well as a strong social support system, among others, all contribute to resilience in adulthood (Feder et al., 2009).

Despite the growing interest in the construct, the implementation of resilience training and monitoring programs has proven difficult, mainly because of the absence of objective measurements of resilience. Presently, the assessment of resilience almost exclusively relies on psychometric tests or interview-based measures. While these approaches have considerable value, subjective assessments are highly susceptible to non-intentional and intentional biases, particularly when participants (e.g., military personnel, police officers, first responders) associate certain responses to future occupational perspectives (Walker et al., 2017). Moreover, resilience may be state- and experience-dependent, therefore it is important to focus on individual differences and underlying processes (Rutter, 2006; Russo et al., 2012). A potential solution to these challenges is the introduction of objectively measurable physiological and/or biological based predictors of resilience, which are less prone to manipulation and subjective bias, offering the possibility to complement existing psychometric scales for improved accuracy in the assessment of resilience. Furthermore, biomarkers of resilience may shed light on underlying neurobiological processes, ultimately leading to the development of novel approaches for the prevention, early detection and timely treatment of psychiatric disorders and psychological distress. Several potential biomarkers of resilience have been proposed (Chen et al., 2015; Daskalakis et al., 2016; Palmfeldt et al., 2016; Walker et al., 2017). Among these, one candidate is vagally-mediated heart rate variability (HRV), which reflects the dynamic modulation of vagal control of the heart rate in response to changing environmental demands. Evidence suggests that vagally-mediated HRV serves as an index of how strongly top-down appraisals, mediated by cortical-subcortical pathways, shape brainstem activity and autonomic responses in the periphery of the organism (Thayer et al., 2012; Gillie and Thayer, 2014). Notably, individuals with high vagally-mediated HRV at rest have been shown to score higher on trait resilience psychometric scales (Souza et al., 2013), whereas chronic reductions in vagal activity indexed by HRV have been consistently associated with psychopathology (Kemp et al., 2010; Chalmers et al., 2014; Gillie and Thayer, 2014; Sgoifo et al., 2015; Clamor et al., 2016).

More recently, neuroscience has addressed the question of the neurobiological mechanisms that might contribute to resilience in humans, which is a key step toward designing effective prevention and treatment programs. The interplay between various hormones, neuropeptides, neurotransmitters, and neural circuits associated with resilience vs. vulnerability to stress-related psychological distress has already been discussed in a number of thorough reviews (Charney, 2004; Karatsoreos and McEwen, 2013; McEwen, 2016; Faye et al., 2017; Osório et al., 2017) and falls beyond the scope of the present paper.

Over the past decades, neuroimaging has become an increasingly important tool to study inter-individual variability in resilience and the development of stress-related psychiatric symptoms. Notably, structural neuroimaging research has shown associations between regional brain morphometric characteristics, particularly cortical thickness, and resting state vagally-mediated HRV (Winkelmann et al., 2017; Yoo et al., 2017). This review is intended to suggest that a multidisciplinary approach integrating structural brain imaging with HRV monitoring could offer novel predictions about brain-body pathways in resilience to psychological stress. The ensuing sections will summarize theoretical views and experimental evidence that support the utility of HRV measures, as surrogate index of resting cardiac vagal modulation, for the prospective assessment of resilience in adulthood. Furthermore, recent structural imaging findings linking individual differences in resting state vagally-mediated HRV to cortical thickness of specific brain surface regions will be introduced. Finally, studies examining structural brain characteristics related to resilience to psychological stress in general and to specific forms of psychopathology will be considered.

## Neural control of cardiac autonomic function and heart rate variability

In order to maintain a fine balance between sympathetic and parasympathetic (vagal) influences on cardiac activity, the autonomic nervous system (ANS) is under the regulation of a complex neural network, allowing the individual to respond accordingly and timely to internal and external demands (Verberne, 1996). An extensive body of research using pharmacological, lesion and neuroimaging techniques both in humans and animals has been directed at identifying the pathways by which neural control of cardiac autonomic function is achieved (Ahern et al., 2001; Critchley et al., 2003, 2011; Gianaros et al., 2004; Lane et al., 2009; Buchanan et al., 2010; Thayer et al., 2012). Taken together, these studies implicate a distributed set of cortical, subcortical, and medullary structures in the regulation of cardiac autonomic function. Coherent and complementary results were obtained in a neuroanatomical retrograde tracing study, where a neurotropic virus was injected into rodent cardiac tissue (Ter Horst and Postema, 1997). These structures form a major part of what is collectively termed the “central autonomic network (CAN)” (Benarroch, 1993) and include: the anterior cingulate, insular, orbitofrontal, and ventromedial cortices; the central nucleus of the amygdala; the paraventricular and related nuclei of the hypothalamus; the periacqueductal gray matter; the nucleus of the solitary tract; the nucleus ambiguous; the ventrolateral medulla; the ventromedial medulla and the medullary tegmental field. A detailed description of the afferent and efferent pathways of this neural network has been comprehensively covered by others (e.g., Benarroch, 1993; Saper, 2002; Palma and Benarroch, 2014). The primary output of the CAN is mediated through the preganglionic sympathetic and parasympathetic neurons, which exert control over the heart via the stellate ganglia and the vagus nerve, respectively. The interplay of sympathetic and parasympathetic influences on sino-atrial node pacemaker activity generates the complex variability that characterizes the healthy heart rate rhythm, which is called HRV (Task Force of the European Society of Cardiology and the North American Society of Pacing and Electrophysiology, 1996). Under resting conditions, vagal influences prevail and HRV is largely dependent on parasympathetic modulation (Task Force of the European Society of Cardiology and the North American Society of Pacing and Electrophysiology, 1996). A fundamental principle of the neural control of the heart is its hierarchical organization, with cortical structures providing inhibitory control over limbic and brainstem sympathoexcitatory, cardioacceleratory circuits (Verberne, 1996; Ahern et al., 2001; Thayer, 2006). Indeed, disruption of prefrontal activity leads to disinhibition of sympathoexcitatory circuits, with a resultant increase in heart rate and decrease in vagally-mediated HRV (Verberne, 1996; Ahern et al., 2001). Furthermore, left-sided (dominant hemisphere) forebrain structures appear to be predominantly involved in vagal regulation, whereas homotopic right (non-dominant) forebrain regions seem to primarily control sympathetic tone and responses (Craig, 2005; Guo et al., 2016). However, the lateralization model of autonomic control of the heart remains controversial (Thayer et al., 2012).

Heart rate variability can be quantified both in the time- and frequency-domains (Task Force of the European Society of Cardiology and the North American Society of Pacing and Electrophysiology, 1996) as well as using more complex techniques based on nonlinear dynamics and chaos theory (e.g., Merati et al., 2004; Porta et al., 2009). In the following sections, we will focus on relevant publications that have quantified vagally-mediated HRV using time-domain indexes such as RMSSD (i.e., the root mean square of successive differences in heart period series) and frequency-domain indexes such as the power in the high frequency (HF) band of the spectrum.

## Psychosocial factors and adult resilience: the link with heart rate variability

Resilience in the context of a specific aversive or stressful experience is considered as a higher-order, multidimensional phenomenon embracing an individual's biological and psychological profile, developmental history, previous traumatic experiences, active choices, social context, current environment, social support, and timing of the stressful event (Charney, 2004; Feder et al., 2009; Cicchetti, 2010). Consequently, defining resilience simply as the absence of psychological symptoms after stressful life experiences does not fully cover the multidimensional nature of this construct. The term resilience as used throughout this review will therefore be more reflective of the ability of an individual to avoid the negative behavioral and biological consequences and cognitive impacts of stressful life events that would otherwise compromise their psychological and physical well-being (Russo et al., 2012).

An earlier review on this topic identified individual characteristics and psychological dispositions that contribute to resilience in adulthood, i.e., optimism, cognitive reappraisal, active coping, humor, social support seeking, prosocial behavior, and trait mindfulness (Wu et al., 2013). Notably, all of these but humor have been associated with high levels of vagally-mediated HRV as illustrated by the examples that follow. Oveis et al. (2009) reported a positive correlation between resting state vagally-mediated HRV and self-reported levels of optimism assessed a month later. Cognitive reappraisal is defined as an effective emotion regulation strategy that implies a cognitive change of the meaning of an emotion-eliciting situation, in order to reduce negative feelings (Gross, 1998). As such, both short-term resting and 24-h vagally-mediated HRV were found to be inversely associated with self-reported difficulties in emotion regulation, particularly the inability to accept negative emotions (Williams et al., 2015; Visted et al., 2017). With regards to adaptive coping strategies, a study on recently bereaved and depressed individuals found that higher levels of resting vagally-mediated HRV were (i) positively associated with measures of active coping and acceptance and (ii) negatively associated with measures of passive coping (O'Connor et al., 2002). Stephen Porges' Polyvagal Theory (Porges, 2007) postulates that higher vagal activity reflected in high HRV underlies prosocial tendencies. This assumption has been empirically supported by both cross-sectional (Porges, 2003; Beffara et al., 2016) and longitudinal studies (Eisenberg et al., 1995). Notably, Bornemann and colleagues further showed that the ability to upregulate HRV via biofeedback predicts individual differences in altruistic prosocial behavior (Bornemann et al., 2016). Moreover, studies have supported the relationship between resting HRV and social support seeking (Kok and Fredrickson, 2010; Geisler et al., 2013). In recent years, growing interest in the construct of trait mindfulness as a psychological factor linked to resilience has emerged. Mindfulness has been defined as “the awareness that arises through intentionally attending in an open, accepting, and discerning way to whatever is arising in the present moment” (Shapiro, 2009), and it can be cultivated by meditation practices derived from Buddhist tradition. Svendsen and colleagues showed that higher levels of trait self-compassion—a construct that encompasses trait mindfulness- were associated with higher short-term resting and 24-h vagally-mediated HRV (Svendsen et al., 2016). Again, it is important to note that increases in vagally-mediated HRV via non-invasive transcranial direct current stimulation (tDCS) or a self-compassion manipulation are associated with increases in soothing emotions more strictly associated with a mindful and compassionate disposition (i.e., relaxed, serene, content, calm, tranquil, peaceful) and ultimately with the construct of resilience (Petrocchi et al., 2017a,b). Interestingly, among the non-pharmacological tools that could be exploited to increase vagally-mediated HRV in low resilient individuals, including tDCS, meditation practices and physical exercise, vagus nerve stimulation (VNS) or transcutaneous VNS is emerging as an innovative approach targeting vagal activity in a variety of somatic and psychological disorders (reviewed in Howland, 2014; Bonaz et al., 2016).

## Vagally-mediated heart rate variability as a physiological marker of stress resilience

Quite surprisingly, despite a rich and growing body of theories and experimental evidence suggesting a strong association between vagally-mediated HRV and psychosocial factors linked to resilience in humans, so far only a few studies have directly investigated the utility of HRV measures as physiological predictors of resilience.

### Recovery from acute psychological stress

HRV and self-reports of resilience were assessed during a laboratory psychosocial stressor (i.e., Trier Social Stress Test, TSST Kirschbaum et al., 1993) in healthy male military personnel after returning from a peacekeeping mission (Souza et al., 2013). The main findings were that participants with higher levels of pre-stress, resting state vagally-mediated HRV (i.e., RMSSD) (i) scored higher on the Ego-Resilience Scale employed in this study, (ii) showed larger tachycardia and HRV reduction during the TSST, and (iii) recovered more efficiently after termination of the stress protocol (as assessed by both heart rate and HRV recovery) (Souza et al., 2013), replicating previous results from the same group (Souza et al., 2007). Likewise, impaired recovery of cardiovascular, endocrine, and immune markers following a mental stress test were found to be associated with lower levels of pre-stress, resting state vagally-mediated HRV (i.e., RMSSD) in *n* = 44 healthy men (Weber et al., 2010). Taken together, these results are in agreement with experimental evidence linking resilience to effective physiological responses during the occurrence of a stressor (al'Absi, 2018) and prolonged post-stress recovery of physiological variables to psychological traits and/or states ascribed to a low-resilient profile. For example, scores on the Ego-Resilience Scale were found to be negatively related to the duration of recovery of six cardiovascular variables (heart rate, finger pulse amplitude, pulse transmission times to the finger and the ear, and systolic and diastolic blood pressure) after a laboratory stressor in adult individuals (*n* = 57, 74% females) (Tugade and Fredrickson, 2004). Notably, prolonged physiological activation after stress exposure has gained recognition as a decisive element in the link between stress and disease (Brosschot et al., 2005). Potential psychological mediators of this association include worry and rumination, or other processes characterized by perseverative cognition, including unconscious processes (Brosschot et al., 2016). Supporting this view, a recent meta-analysis revealed associations between perseverative cognition and elevated systolic and diastolic blood pressure, heart rate, and cortisol, as well as reduced HRV (Ottaviani et al., 2016).

### Resilience to psychological and somatic distress

Chronic reductions in resting state HRV are associated with a wide range of psychological disorders, including PTSD (e.g., Agorastos et al., 2013; Gillie and Thayer, 2014), depression (e.g., Rottenberg, 2007; Kemp et al., 2010; Sgoifo et al., 2015), and anxiety disorders (e.g., Chalmers et al., 2014; Makovac et al., 2016) among others. Therefore, reduced HRV is conceived as an autonomic, transdiagnostic biomarker of psychopathology (Beauchaine and Thayer, 2015). However, an important limitation of these prior studies is that associations between HRV and psychopathology have been explored cross-sectionally only, by comparing data obtained from psychiatric patients and matched healthy controls. Consequently, one unresolved causal/temporal issue in resilience research is whether differences in HRV found between psychiatric patients and healthy subjects are a result of stress exposure or are a characteristic of the low-resilience phenotype that was already present before the stressful events. Prospective studies that correlate pre-stress measurements with subsequent development of psychological symptomatology have just started addressing this question. The Marine Resilience Study (Minassian et al., 2015) evaluated resting HRV 1–2 months before a combat deployment in *n* = 2160 young male Marines, *n* = 83 of whom met PTSD diagnostic criteria 4–6 months after returning. Overall, the results indicated that lower resting HRV at pre-deployment assessment, as measured by a higher low frequency (LF):HF ratio (i.e., a controversial measure of sympathovagal balance), was associated with increased risk of a PTSD diagnosis after deployment, after taking several key risk factors such as age and deployment-related traumatic brain injury among others into account (Minassian et al., 2015). A potential limitation of this study is that the reliability of the LF:HF ratio as a robust and specific measure of sympathovagal balance has been criticized, particularly in situations when respiratory rate is not accounted for (Billman, 2013; see for discussion Heathers and Goodwin, 2017). Moreover, no significant associations were observed between post-deployment PTSD and pre-deployment vagally-mediated HRV (i.e., the HF component) (Minassian et al., 2015). The authors concluded that a small sample size for the PTSD group may have reduced the power to detect these potential associations (Minassian et al., 2015). Nevertheless, this prospective longitudinal study provided initial evidence of the utility of resting state HRV measures for the prediction of PTSD vulnerability. The subsequent Warriors Achieving Resilience study (Pyne et al., 2016) examined the relationship between pre-deployment resting HRV and 3- and 12-month post-deployment PTSD symptom severity in *n* = 343 Army National Guard soldiers. The results indicated that pre-deployment resting state vagally-mediated HRV (i.e., the HF component) was a significant predictor of continuous and dichotomous PTSD symptom severity, but only for those soldiers with higher pre-deployment PTSD symptoms, after controlling for several covariates (Pyne et al., 2016). The authors acknowledged several limitations of this study, including the assessment of pre-deployment HRV during a time of stressful training and the use of the PTSD Checklist - Military version as the outcome measure, which soldiers recognize as a PTSD screening questionnaire and as such is susceptible to intentional bias (Warner et al., 2011; Sundin et al., 2014). Notwithstanding these limitations, this longitudinal study suggests that pre-deployment vagally-mediated HRV may represent a significant physiological predictor of post-deployment PTSD symptom severity in the context of higher levels of pre-deployment PTSD symptomatology.

A recent longitudinal study assessed resting state HRV and depressive symptoms in *n* = 42 non-clinical young individuals (52% women) over a time span of almost 3 years (Carnevali et al., 2017b). The main findings were that vagally-mediated HRV (i.e., RMSSD) during resting conditions (i) predicted changes in depressive symptoms over the 3-year period, and (ii) was a significant mediator of the positive association between perseverative cognition processes (i.e., rumination) and depressive symptomatology (Carnevali et al., 2017b). Even though the clinical impact of these findings might be questionable given the relatively small sample size, and despite the fact that the authors did not control for the occurrence of stressful life events before the assessment points, these results reinforce the idea that resting state vagally-mediated HRV could represent a useful physiological marker of resilience to stress-related psychological disorders, including depression. Supporting this view, in a large longitudinal population-based cohort study (the sample size ranged from *n* = 2334 (*n* = 644 women) to *n* = 2276 (*n* = 602 women) depending on the analysis), lower baseline heart rate and higher vagally-mediated HRV were found to be associated with a lower likelihood of incident depressive symptoms over a 10-year interval in men without depressive symptoms at baseline. Similar but statistically insignificant associations were found in women (Jandackova et al., 2016). Notably, lowered HRV is also a widely recognized prognostic risk factor for somatic disease, including cardiovascular disorders (Thayer et al., 2010), potentially playing a role in the still unexplained association between depression and increased cardiovascular risk (Rottenberg, 2007; Sgoifo et al., 2015). Another proposed mechanism by which negative emotion can confer risk for cardiovascular disorders involves exaggerated cardiovascular reactivity to psychological stress; the major principle of the reactivity model is that individuals with larger cardiovascular reactivity have higher risk of cardiovascular morbidity (e.g., Barnett et al., 1997; Treiber et al., 2003). However, a quantitative review revealed that cardiovascular (heart rate and blood pressure) responses to laboratory stressors are only moderately related to depressive symptoms (Kibler and Ma, 2004). Moreover, patients with PTSD were found to show higher heart rate and blood pressure increases and larger HRV decreases specifically in response to trauma-related tasks (Blanchard et al., 1982, 1996; Keary et al., 2009). However, lack of consistency was found in cardiovascular responses to trauma-unrelated stressors: while some studies reported no difference between PTSD patients and healthy controls in cardiovascular changes induced by a mental arithmetic or the cold pressor test (Orr et al., 1998; Jones-Alexander et al., 2005), other researchers reported that PTSD was associated with reduced cardiovascular reactivity in response to trauma-unrelated stressors (Keane et al., 1998; McDonagh-Coyle et al., 2001). Therefore, poor cardiovascular recovery following psychological stress might be more relevant for the development of stress-related pathology than stress reactivity itself, as further suggested by a recent meta-analysis (Panaite et al., 2015). Notably, a large population-based longitudinal study found that cardiovascular (blood pressure and heart rate) responses to acute psychological stress were negatively associated with depressive symptomatology 5 years later (Phillips et al., 2011).

In summary, despite the small number of studies that have explicitly examined the predictive power of HRV measures as physiological markers of resilience (and despite some obvious limitations for each of them), a trend is becoming evident. It appears that individuals who show higher vagally-mediated HRV during resting conditions rate themselves as having higher scores in the resilience questionnaires, recover more efficiently from acute psychological stress, and are less vulnerable to the development of PTSD- and depression-related symptomatology.

## Brain structural correlates of individual differences in heart rate variability

In the last several years, the rapid growth of modern neuroimaging techniques has enabled researchers to study the neural correlates of HRV. Several groups have attempted to relate estimates of HRV with concurrent brain response using positron emission tomography (PET) (Gianaros et al., 2004; Ahs et al., 2009; Lane et al., 2009) or functional magnetic resonance imaging (fMRI) (Critchley et al., 2003; Napadow et al., 2008) during behavioral or dynamic exercise tasks that are thought to perturb ANS activity. Other groups have assessed correlations between fMRI task responses with out-of-scanner baseline HRV (Neumann et al., 2006; O'Connor et al., 2007) or HRV response to the in-scanner task (Matthews et al., 2004). These studies were included in a meta-analytic review (Thayer et al., 2012) which identified significant associations between vagally-mediated HRV and cerebral blood flow in the right pregenual and right subgenual anterior cingulate cortices and in the left sublenticular extended amygdala/ventral striatum. On the other hand, an explicit investigation of the brain structural correlates of HRV has just started to emerge.

### Brain morphology and heart rate variability in healthy adults

**Figure 1:**
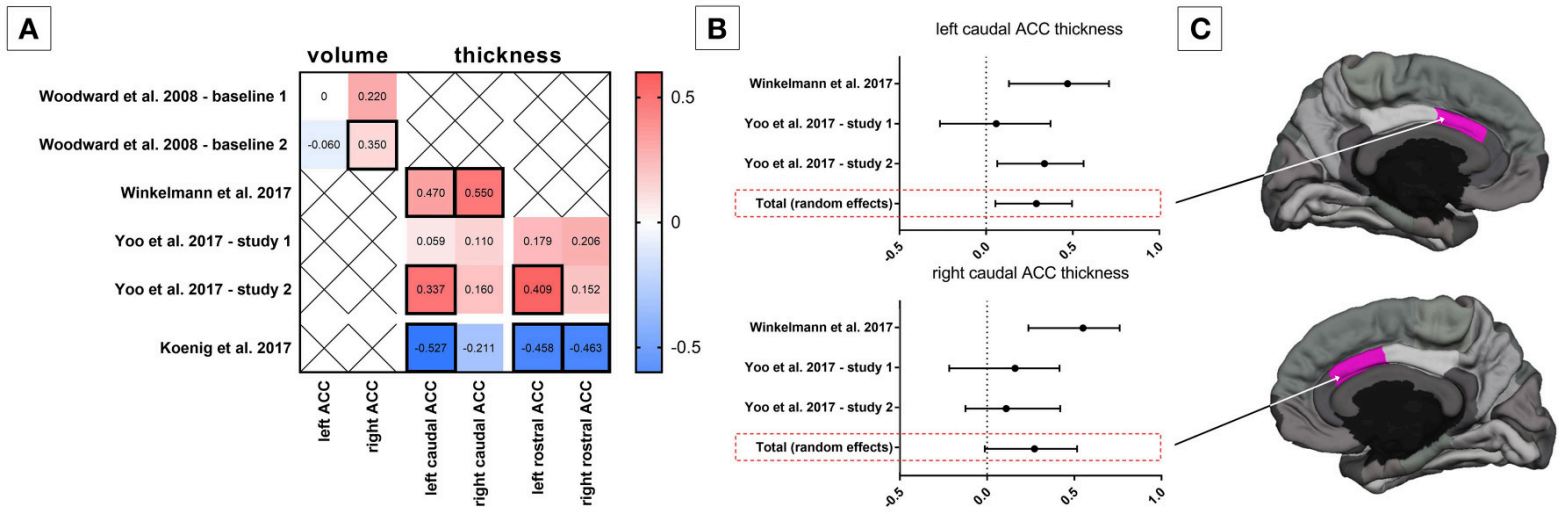
Association between heart rate variability (HRV) and anterior cingulate cortex (ACC) morphology. **(A)** Correlation coefficients between HRV and ACC volume and structural thickness from existing studies; statistical significant correlation coefficients are framed by a thick line. **(B)** Mini meta-analysis (random effects model) on correlation coefficients between HRV and caudal ACC structural thickness in adults. **(C)** Schematic illustration of the caudal ACC in the hemisphere medial view.

The first investigation of the link between HRV and brain morphology dates back to 2008 (Woodward et al., 2008). Using a region of interest (ROI) analysis of manually traced anterior cingulate cortex (ACC) volumes, it was found that the magnitude of respiratory sinus arrhythmia (i.e., an ECG-based marker of cardiac vagal modulation in synchrony with respiration) during resting conditions was positively associated with the right but not left ACC volume in *n* = 77 predominately male U.S. combat veterans (mean age 49 years), independently from the presence of a diagnosis of PTSD (Woodward et al., 2008) (Figure 1). Subsequently, the study of the structural brain correlates of HRV has been approached using other morphometric measures such as cortical thickness, which is suggested to be a more sensitive parameter with a higher signal-to-noise ratio (Dickerson et al., 2008; Hutton et al., 2009) and more easily interpretable than the probabilistic gray matter volumes in voxel-based morphometry (Lehmann et al., 2011). It must be noted, however, that cortical thickness measures only permit evaluation of the cortical surface, and thus do not allow analysis of deeper structures including the amygdala or even the hippocampus. Moreover, the development of automated segmentation tools allows for increasingly reliable and accurate whole-brain segmentation and quantification of cortical morphology (e.g., Fischl, 2012). Using these techniques, a recent study showed that the resting state vagally-mediated HRV (i.e., the HF component) was positively associated with cortical thickness of the caudal ACC (Figure 1), more strongly with the right side, in a group of *n* = 30 young participants (mean age 22.53 ± 3.89 years, 8 females) (Winkelmann et al., 2017) (Figure 1). Additional positive, but weaker, associations were found between vagally-mediated HRV and cortical thickness of the superior frontal gyrus, pars triangularis, the rostral middle frontal gyrus, the precentral gyrus and the lingual gyrus, all in the right hemisphere (Winkelmann et al., 2017). One major limitation of this study is the small sample size, which precluded the investigation of potential sex- and age-related differences. A very recent study in a large sample of healthy subjects (*n* = 185 (95 females), mean age 35.2 ± 14.0 years) identified significant negative relationships between resting state vagally-mediated HRV (i.e., the HF component) and gray matter volumes in a number of striatal and limbic regions, including the right putamen, caudate, amygdala, insula, superior temporal gyrus, temporal pole, and parahippocampal gyrus (Wei et al., 2018). Of note, these associations did not vary significantly as a function of sex and/or age (Wei et al., 2018). Although the cross-sectional design of this study does not allow drawing any general inference about the causal mechanisms underlying the association of gray matter volume with cardiac vagal activity, these findings emphasize the importance of striatal and limbic structures in the neural control of HRV.

### Age and sex dependent findings

Ageing is known to be linked both to decreases in HRV (Antelmi et al., 2004; Zhang, 2007) and substantial brain structural changes (Peters, 2006; Fjell et al., 2009; Fjell and Walhovd, 2010). The influence of aging on the association of resting state vagally-mediated HRV (i.e., RMSSD) with cortical thickness was explicitly investigated in two independent samples (Yoo et al., 2017). The first sample included *n* = 21 older adults (10 males, age-range 61–78) and *n* = 20 younger adults (12 males, age-range 19–37), while the second sample comprised *n* = 28 older adults (16 males, age-range 55–75) and *n* = 35 younger adults (21 males, age-range 18–34). Several noteworthy findings emerged from a meta-analysis of the results of these two samples. Similar to the previous report with a separate group of young adults (Winkelmann et al., 2017), the cortical thickness of the caudal ACC was found to be positively correlated with resting state vagally-mediated HRV (Yoo et al., 2017) (Figure 1). However, in this study the left sided association was stronger and the right-sided association did not reach the set level of significance (Yoo et al., 2017) (Figure 1). Furthermore, a significant positive correlation was found between left rostral ACC cortical thickness and vagally-mediated HRV (Figure 1). Notably, the association of vagally-mediated HRV with ACC cortical thickness appeared to change with age, as the correlations were weaker in models adjusting for age (Yoo et al., 2017). Similarly, another study identified an age-dependent positive relationship between cortical thickness of the medial prefrontal cortex (mPFC) and vagally-mediated HRV (i.e., the HF component) in a sample of *n* = 55 healthy individuals (21–73 years; 18 females) (Wood et al., 2017). On the contrary, an age-invariant association was identified between HRV and cortical thickness of the left lateral orbitofrontal cortex (OFC) (Yoo et al., 2017). This may be explained by the fact that ventral regions such as the lateral OFC seem to be less susceptible to age-related decreases in cortical thickness compared to more superior, dorsal and lateral regions (Fjell et al., 2009; Mather, 2016). Indeed, besides the ACC and mPFC, several other regions such as the caudal (both hemispheres) and rostral (right hemisphere) middle frontal gyrus, pars opecularis (both hemispheres), pars orbitalis (right hemisphere), pars triangularis (both hemispheres), superior frontal gyrus (both hemispheres) and the insula (both hemispheres) were identified as being associated with HRV, but only when younger and older adults were included as one group (Yoo et al., 2017). Unfortunately, a limitation of this study (Yoo et al., 2017) is inherent in its cross-sectional design, which may bias results due to potential cohort differences. Consequently, future longitudinal studies are needed to more definitively examine whether cortical thinning of specific brain regions, including the ACC, with age may contribute to the age-related decline in HRV. A very recent study aimed to further explore age-related changes in the brain structural concomitants of HRV. In a small sample of female adolescents (*n* = 20, mean age 15.92 years), associations between vagally-mediated HRV and the right caudal ACC and the rostral ACC (independent of hemisphere) were confirmed (Koenig et al., 2017a) (Figure 1). However, unlike in adults, adolescents show an inverse association between cortical thickness of the ACC and vagally-mediated HRV, suggesting that (i) greater vagally-mediated HRV is beneficial for cortical development during adolescence (i.e., cortical thinning) or that (ii) cortical thinning contributes to pubertal changes in vagally-mediated HRV, which increases up to early adulthood and thereafter decreases with increasing age.

It is critical to note that important sex differences have been reported in cortex morphology (Barnes et al., 2010). Specifically, females have been shown to have a thicker cortex across many regions of the brain than males, more evidently in the temporal and parietal lobes of the right hemisphere (Luders et al., 2006; Sowell et al., 2007). Moreover, adult women have generally been reported to show increased vagally-mediated HRV relative to men (Koenig and Thayer, 2016), while in underage samples vagally-mediated HRV is decreased in girls compared to boys (Koenig et al., 2017b). Whether sex-specific morphological characteristics of the brain influence neural control of HRV differently in females than males and how this relationship varies as a function of age is yet to be explicitly investigated. However, similarly to the above presented reports in mixed-gender populations, cortical thickness of the left caudal ACC was recently found to be positively associated with resting state vagally-mediated HRV (i.e., RMSSD) in a small sample of young females (*n* = 18, mean age 27.2 ± 1.7 years) (Carnevali et al., submitted).

In summary, despite only a handful of studies exploring the brain structural correlates of HRV, and despite some limitations of individual studies, morphological aspects of several prefrontocortical, striatal and limbic regions appear to be associated with resting state vagally-mediated HRV, aiding our efforts to better understand the underlying anatomical basis of parasympathetic autonomic modulation. Furthermore, the most consistent finding across studies and sexes is that ACC cortical thickness is positively correlated with resting state vagally-mediated HRV in healthy young adults. This region has been recognized as the one most related to HRV also in functional studies (Thayer et al., 2012). The association between ACC cortical thickness and HRV seems to become weaker with increasing age and seems to be reversed in underage samples. This might be due to an age-related thinning of this brain region and may account for the decline in HRV observed with adult aging.

## Structural neuroimaging of resilience

The observation that in healthy individuals resting vagally-mediated HRV may be related to specific regional brain morphological characteristics raises an intriguing question: are the brain structural aspects of resilience similar to those implicated in the expression of individual differences in vagally-mediated HRV? If so, this would strengthen the idea that resting state vagally-mediated HRV could be a used as a reliable, easily measurable, physiological predictor of resilience.

Within the past several years, a growing number of brain imaging studies in humans and animals have been directed at delineating the brain circuits that mediate distinct aspects of emotion and stress regulation during normal psychological functioning and various psychopathological conditions that are indicative of low resilience. Key structures involved in this neurocircuitry are the amygdala, insula, hypothalamus, hippocampus, and cortical structures such as the mPFC and the ACC (for an extensive literature on the subject the reader is referred to Feder et al., 2009; Franklin et al., 2012; Wu et al., 2013). However, despite the rapid growth of modern neuroimaging techniques, research into the regional brain morphological correlates of resilience is still very limited. Ideally, brain structural aspects of resilience should be studied longitudinally, by performing measurements before and after stressful events and comparing the differences between those who resisted the psychological impact (i.e., resilient) and those who developed psychological disorders. Such prospective investigations are lacking in humans. Current insights in this area stem mostly from cross-sectional studies, in which stress-exposed psychiatric patients, frequently with PTSD, are compared to individuals with no symptoms of psychopathology, and as such are considered as resilient. However, many of these studies did not include a healthy, non-exposed control group, which is crucial in cross-sectional designs to establish whether differences between exposed groups are related to vulnerability or resilience. Despite these obvious limitations, structural studies point to larger gray matter volumes in structures such as the ventral mPFC, the rostral ACC and the subgenual ACC in resilient individuals (van der Werff et al., 2013). These regions have been implicated in the modulation of emotional responsiveness through top-down control over the amygdala and may represent an important neural substrate underlying more efficient stress responses in resilience (Phelps et al., 2004; Baumann and Turpin, 2010). On the other hand, individuals with low resilience may have compromised cortico-limbic inhibition, making them more vulnerable to stress-related disorders. Indeed, hypofunction of the PFC, the rostral ACC and the subgenual ACC, and hyperactivity of amygdala have been related to dysregulation of emotion in anxiety and mood disorders (Phan et al., 2005; Drevets et al., 2008a,b; Shin and Liberzon, 2010). Moreover, previous studies have identified reduced volumes (Rauch et al., 2003; Yamasue et al., 2003; Woodward et al., 2006) and abnormal shape (Corbo et al., 2005) of the ACC in patients with PTSD, and found that these morphological measures were correlated with PTSD symptom severity scores. Smaller gray matter volumes of the ACC and mPFC have also been reported in patients with depression compared to healthy controls (Drevets, 2001; Drevets et al., 2008a,b). Furthermore, abnormal structural integrity (cortical thinning) of the ACC has been implicated in the expression of autonomic dysfunction (reduced HRV) in a small sample of young female patients with generalized anxiety disorders (*n* = 17, mean age 30.7 ± 2.0 years) (Carnevali et al., submitted). However, a study on twins (Vietnam veterans exposed to trauma vs their twins who were not exposed) showed that gray matter volume reductions in the ACC were not present in non-PTSD co-twins, suggesting that reduced volume of the ACC could be the consequence of the exposure to stress, rather than a predisposing risk factor (Kasai et al., 2008). On the other hand, evidence of the association between structure of the amygdala or the hippocampus, key regulatory structures in the stress response, and resilience as well as PTSD symptomatology is currently inconsistent and inconclusive (Morey et al., 2012; van der Werff et al., 2013).

A recent study investigated resilience-related morphological differences in brain regions involved in cortico-limbic inhibition in a sample of *n* = 48 young individuals (mean age 26.31 ± 6.96 years, 33 females) without any previous or current major psychiatric or medical disease (Gupta et al., 2017). Notably, a positive association was found between cortical thickness of the ACC and resilience scores, particularly those related to the bounce-back subscale of the Connor-Davidson Resilience questionnaire (Gupta et al., 2017). Moreover, subjective measures of resilience were found to be significantly associated with greater gray matter volume of the right amygdala (Gupta et al., 2017). The authors speculated that reduced volume of the amygdala associated with low resilience may develop in individuals with compromised inhibitory control (Gupta et al., 2017).

As discussed above, coping styles may serve as a mediator between stress exposure and psychopathology (Feder et al., 2009). A recent study found that positive coping styles were associated with increased ACC volume, specifically with the perigenual extending to the subgenual part, in a large sample (*n* = 106) of 22 year-old females (Holz et al., 2016). Notably, in this study both ACC volume and positive coping styles predicted symptoms of anxiety and depression over a 3 year-time span in a sex-dependent manner, with inverse relationships emerging in females only (Holz et al., 2016). The authors argued that protective factors such as increased positive coping styles might emerge only when stress-reducing mechanisms are needed and that studies in clinical samples with a higher stress load across both sexes may not replicate a sex-dependent effect, but may rather confirm effects of positive coping styles and ACC volume on psychopathology for both sexes (Holz et al., 2016).

In sum, despite the limited availability of studies that have explicitly investigated regional brain morphological correlates of resilience, structural aspects of the ACC appeared to be closely interlinked with psychological traits ascribed to a high-resilient profile. In particular, when viewed together with the abnormal structural integrity of the ACC in patients with PTSD or depression, associations between ACC cortical thickness/volume and resilience scores/positive coping styles suggest that morphological aspects of the ACC may be responsible for a greater ability to engage feedback inhibition of the amygdala, limiting the extent, and duration of stress circuit activations in high resilient individuals.

## Conclusion

Individuals with enhanced stress resilience mechanisms have the ability to adapt successfully to life stress without developing persistent psychopathology. The identification of physiological predictors of resilience as well of the potential mediators on different levels (i.e., psychological, neural, (epi)genetic, etc.) is a major goal of resilience research. This review aimed to integrate theoretical views and empirical evidence suggesting that individual differences in resting state vagally-mediated HRV may underlie differential resilience to the development of stress-related psychological disorders. Notably, positive associations between morphological characteristics of the ACC, particularly its cortical thickness, and individual differences in vagally-mediated HRV have now been reported in several independent studies. Structural neuroimaging, including MRI, has also contributed significantly to our understanding of the morphological changes associated with specific forms of psychopathology. Abnormal structural integrity of brain regions involved in cortico-limbic inhibition, including the mPFC and the ACC, has been described in PTSD (Rauch et al., 2003; Yamasue et al., 2003; Woodward et al., 2006), depression (Drevets, 2001; Drevets et al., 2008a,b), and anxiety disorders (Shang et al., 2014). However, as most research in humans on the aforementioned topics is cross-sectional, no causal conclusions can yet be drawn on the temporal order between stress exposure and brain structural changes observed in psychiatric patients. Moreover, changes in the brain associated with psychiatric illness may occur much earlier than observable symptoms (Insel, 2013). To date, a direct examination of the morphological brain correlates of resilience is absent, and empirical work in this area remains sparse. Nevertheless, the few available studies point to positive associations between morphological aspects of the ACC (cortical thickness and volume) and trait resilience or coping strategies known to facilitate the adaptation to stressful situations (van der Werff et al., 2013; Holz et al., 2016; Gupta et al., 2017). Volume and thickness are linked to each other by a simple mathematical equation, the volume being the product of the thickness of the cortical mantle by the surface area. However, it is critical to note that these morphometric measures may not be equally sensitive to factors associated to cortical atrophy, such as aging, or may be differentially affected by developmental history and/or previous (traumatic) experiences, and thus may present their own specificity. Nevertheless, compromised structural integrity of the ACC might represent an important substrate of reduced HRV in several forms of psychopathology (Beauchaine and Thayer, 2015). Similarly to the point we have raised above, the cross-sectional nature of most available research in humans does not allow to clarify whether differences in HRV between psychiatric patients and healthy controls represent a biological diathesis, a compensatory adaptation, or a consequence of the illness. Based on our present review, we would hypothesize that lower vagally-mediated HRV is a characteristic of the low resilient phenotype that was already present before the stressful event and is linked to cortical thinning of the ACC in adults. Hence, reduced cortical thickness of the ACC and low resting state vagally-mediated HRV might serve as high-risk endophenotypes for the development of psychopathology. However, at present there is no evidence on the causal relationship between cortical thickness and vagally-mediated HRV (i.e., cortical thinning causing reduced vagally-mediated HRV or a decline in vagally-mediated HRV causing reduced cortical thickness), mostly due to the lack of longitudinal studies that have specifically addressed this issue.

In conclusion, we suggest that a multidisciplinary approach integrating brain structural imaging with HRV monitoring could offer novel predictions about brain-body pathways in resilience to psychological stress and might improve the knowledge of etiology, prognosis and treatment of stress-related disorders, which represent a tremendous burden both for individuals and for society as a whole. Ideally, HRV and morphological brain correlates of resilience should be assessed longitudinally, before and after an individual has been exposed to a severe stressful event. Subsequently, differences should be compared between individuals who develop persistent stress-related psychopathology, individuals who do not develop psychopathology, and a control group that has not been exposed to stress. This would allow the identification of HRV measures as baseline predictors of resilience and examine their relationship with morphological aspects of the brain, particularly of the ACC. While this prospective approach may be challenging in most human studies, it could be more easily addressed in studies employing relevant and translational animal models (Beery and Kaufer, 2015; Carnevali et al., 2017a; Sillivan et al., 2017).

## Author contributions

All authors (LC, JK, AS, CO) have contributed to this review; LC has drafted the manuscript; JK, AS, and CO provided intellectual contributions in commenting and revising the manuscript.

### Conflict of interest statement

The authors declare that the research was conducted in the absence of any commercial or financial relationships that could be construed as a potential conflict of interest.
